# Plant-Mediated Behavioural Avoidance of a Weevil Towards Its Biological Control Agent

**DOI:** 10.3389/fpls.2022.923237

**Published:** 2022-06-23

**Authors:** Morgan W. Shields, Steve D. Wratten, Craig B. Phillips, Chikako Van Koten, Stephen L. Goldson

**Affiliations:** ^1^Bio-Protection Research Centre, Lincoln University, Lincoln, New Zealand; ^2^Weeds, Pests and Biosecurity Group, AgResearch Ltd., Lincoln, New Zealand; ^3^Knowledge and Analytics Science Group, AgResearch Ltd., Lincoln, New Zealand

**Keywords:** biological control of insect, host plant effect, *Lolium multiflorum*, *Lolium perenne*, parasitoid, pasture, ploidy, rapid evolution

## Abstract

New Zealand pastures largely comprising *Lolium* ryegrass species (Poales: Poaceae) are worth $19.6B and are subject to major pest impacts. A very severe pest is the Argentine stem weevil *Listronotus bonariensis* (Kuschel) (Coleoptera: Curculionidae). This has been previously suppressed by the importation biological control agent, *Microctonus hyperodae* Loan (Hymenoptera: Braconidae). However, this suppression has recently declined and is subject to investigation. It has been hypothesised that grass type influences the parasitism avoidance behaviour by the weevil and thus parasitism rates. This study explored the hypothesis using three common pasture grasses: a diploid *Lolium perenne* x *Lolium multiflorum* hybrid ryegrass (cv. Manawa), a tetraploid Italian ryegrass *L. multiflorum* Lam. (cv. Tama), and a diploid perennial ryegrass *L. perenne* L. (cv. Samson). The described laboratory-based microcosm methodology determined the extent of weevil avoidance behaviour on each of these three grasses when subjected to the parasitoid. Such reaction was gauged by the extent of reduced weevil on-plant presence and feeding compared to the control populations. In the absence of the parasitoid, the hybrid cv. Manawa ryegrass is as highly favoured by the weevil as the tetraploid cv. Tama. On diploid cv. Samson, feeding is considerably less. In the presence of the parasitoid, weevils on the tetraploid cv. Tama plants showed little avoidance activity in response to the parasitoid and it can be argued that the benefits of staying on this plant outweighed the possibility of parasitism. Conversely and surprisingly, in the parasitoid’s presence, weevils on diploid cv. Manawa showed very strong avoidance behaviour leading to levels of exposure similar to those found on the less-preferred diploid cv. Samson. These findings reflect how weevil parasitism rates have declined in most *Lolium* grasses, particularly diploids, since the 1990s, but not in the tetraploid *L. multiflorum*. This contribution supports the hypothesis that the decline in weevil parasitism rates has been the result of rapid evolution arising from parasitoid-induced selection pressure and the countervailing effect of the nutritional quality of the host plants.

## Introduction

This contribution describes an atypical tri-trophic relationship, whereby congeneric host plants variably influence a pest’s parasitoid avoidance behaviour when in the presence of its natural enemy. The interaction comprises *Loilium* ryegrass (Poales: Poaceae), the Argentine stem weevil *Listronotus bonariensis* (Kuschel) (referred to hereafter as *L. bonariensis* or ‘the weevil’), and its thelytokous parasitoid *Microctonus hyperodae* Loan (Hymenoptera: Braconidae) (referred to hereafter as *M. hyperodae* or ‘the parasitoid’).

*Listronotus bonariensis* is the most serious pest of Gramineae in New Zealand’s improved pastures, causing NZD $200 million worth of damage per annum (Ferguson et al., 2019). The species was first described in New Zealand in 1927 (Marshall, 1937) but it probably arrived far earlier (Stewart et al., 2022). Its biology has been extensively described (e.g., Barker et al., 1989; Goldson et al., 2011). The weevil overwinters in diapause with up to three generations in spring to autumn (Goldson et al., 2011). Significantly, the pasture damage is caused by the stem-mining habit of the four larval instars. The koinobiontic solitary parthenogenetic parasitoid *M. hyperodae* also undergoes a similar number of generations. This parasitoid is host specific (Goldson et al., 1992), is considered to have a high searching efficiency (Tomasetto et al., 2018), and probably uses olfactory cues to search for unparasitised hosts. The Gramineae most damaged by the weevil typically comprises three grass types. The least susceptible is the widely distributed and ubiquitous diploid perennial ryegrass *Lolium perenne* L., e.g., Samson, then diploid hybrid ryegrass (*L. perenne* x *L. multiflorum*), e.g., Manawa, and the most susceptible but relatively uncommon is tetraploid Italian ryegrass, *Lolium multiflorum* Lam, e.g., Tama (Goldson, 1982; Barker, 1989).

The parasitoid *M. hyperodae* was imported from South America in the early 1990s for biological control of *L. bonariensis* and was released at various sites throughout New Zealand (Goldson et al., 1990, 1993; McNeill et al., 2002). Ensuing high parasitism rates of up to 90% in the adults in the common *L. perenne* pastures (Barker and Addison, 2006) were subsequently shown to have had an appreciable impact on the weevil populations (Barker, 2013). However, it was against this background of significant success that mean parasitism rates declined from ca. 70 to 30% after ca. 14 generations (Tomasetto et al., 2017c) with the concurrent reappearance of the pest’s outbreaks (Popay et al., 2011). Goldson et al. (2015) suggested that this was possibly the result of selection favouring enhanced parasitoid avoidance behaviour by the weevil, and it was posited this selection was facilitated by a lack of complexity in New Zealand’s pastures (Goldson et al., 2014) combined with an unequal evolutionary arms race with the weevil being sexual and the parasitoid parthenogenetic (e.g., Goldson et al., 2014; Casanovas et al., 2018). While resistance in importation biological control is very rare (e.g., Pennisi, 2017), a similar evolutionary response was found by Pascoal et al. (2014) in Hawaii, who observed that male crickets (*Teleogryllus oceanicus* Le Guillou) had evolved to become non-stridulating twice *via* different mutation events to avoid detection by a parasitoid fly (*Ormia ochracea* Bigot).

Recent field and laboratory work in New Zealand has shown that weevil parasitism levels across the diploid *Lolium* pasture grasses have undergone a marked reduction between the 1990s and 2015 onwards independent of weevil population density (Goldson and Tomasetto, 2016; Tomasetto et al., 2017c; Goldson et al., 2020, 2022). However, the nationwide decline was not found in the less-common and short-lived tetraploid *L. multiflorum* pastures, perhaps because the weevils had undergone less selection pressure on this less-common grass. That parasitism levels remained unchanged from the 1990s suggests that whatever mechanism leads to reduced parasitism in diploid grasses does not operate in tetraploid *L. multiflorum* grasses (Goldson et al., 2015; Goldson and Tomasetto, 2016; Tomasetto et al., 2017a). Notably, hybrid cultivars showed the same extent of parasitism decline as *L. perenne* pastures (Goldson and Tomasetto, 2016) despite being favoured as hosts by the weevil (Goldson, 1982; Barker, 1989).

Given the increasing understanding of the observed laboratory and field patterns of parasitism decline, this contribution examines in more detail how grass cultivars affect weevil parasitoid-avoidance behaviour. It has been hypothesised that the variation in parasitism decline on the different *Lolium* grasses was due to either enhanced parasitoid-avoidance behaviour by reducing the weevil’s exposure or effects of plant architecture (Goldson et al., 2015). It has since been shown that plant orientation (vertical vs. horizontal placement of tillers) has little effect on parasitism rates (Goldson and Tomasetto, 2016). Shields (2019) conducted a microcosm-based investigation which suggested that parasitoid-avoidance behaviour in the form of reduced feeding and on-plant presence could have contributed to the widespread decline in parasitism rates. This contribution seeks to provide insight into how this happens through plant-mediated effects.

## Materials and Methods

### Grass Selection

The *Lolium* cultivars used in the experiments are New Zealand’s main pasture grasses and have long been used in investigating pasture interactions *with L. bonariensis* and *M. hyperodae* (e.g., Kelsey, 1958; Pottinger, 1961; Goldson, 1982; Barker, 1989; Goldson et al., 2015; Goldson and Tomasetto, 2016). These grasses were diploid hybrid ryegrass cv. Grasslands Manawa (*L. perenne* x *L. multiflorum*) (hereafter referred to as Manawa), tetraploid Italian ryegrass, *Lolium multiflorum* cv. Grasslands Tama (hereafter referred to as Tama), and diploid perennial ryegrass *Lolium perenne* cv. Grasslands Samson (hereafter referred to as Samson). For simplicity, the cultivars used were endophyte free.

### Insect Sampling and Laboratory Maintenance

Adult *L. bonariensis* were collected on 8 January 2018 at Lincoln (43.64397*^o^*S, 172.44292*^o^*E) during the peak emergence of the first summer generation adults (late December to early February) when parasitism rates were low (Goldson et al., 1998; Phillips and Kean, 2017). This timing reduced the probability of weevil-parasitoid interactions prior to the experiment. As determined by availability, the weevils were maintained in cages at an artificially reversed 16:8 light:dark photoperiod, 60% humidity, and ca. 23°C prior to the experiment. Bouquets of Tama were replaced twice a week as food. Prior to the experiment, the weevils were starved for three days but were provided with water-soaked dental wicks.

Weevils were collected again at Lincoln in late February and early March 2018 from which parasitoids were reared. At this time high field parasitism rates occur (Goldson et al., 1998; Phillips and Kean, 2017). The samples were maintained in the conditions described above pending prepupal *M. hyperodae* emergence. Pupae were kept in Petri dishes with water-soaked dental wicks to maintain high humidity. Parasitoid adults were kept in Petri dishes in the same ambient conditions and provided with dental wicks soaked with a 10% honey solution (Phillips et al., 1996). All adult parthenogenetic parasitoids used in the experiments were ≤ 5 days old.

### Plant-Mediated Behavioural Experiment Protocol

An arena configuration was used to measure *L. bonariensis*’ behavioural responses to *M. hyperodae* on the three different host grasses (Manawa, Tama, and Samson). Each grass had a non-parasitoid control. This six-treatment experiment had a randomised block design with 15 replicates.

Each arena comprised an upper section of a transparent polyvinyl chloride jar (230 mm × 12 mm × 8 mm) inserted 20 mm into a 165 mm diameter plant pot containing pasteurised and moistened immature pallic soil (Landcare Research Ltd. 2016). To prevent the weevils from climbing the arena’s sides, polytetrafluoroethylene was added to the lower internal surface, and each arena was capped with.1-mm mesh for ventilation. Twenty-four hours before the experiment, four-week-old plants cropped to 150 mm and comprising 5-10 tillers were planted into each arena. On the day of the experiment, 2 h prior to the onset of darkness, 10 adult weevils were added to each arena, and 90 min later a single parasitoid was added to the non-controls.

For logistical reasons, the behavioural data were collected through direct visual observation on 30 March 2018 (5 blocks), 7 April 2018 (5 blocks), and 12 April 2018 (5 blocks) based on availability of parasitoids ≤5 days old. Each block consisted of one replicate of each treatment. The data from each treatment were analysed together regardless of which block they were recorded from. Data collection commenced at the onset of darkness and was conducted during four consecutive 40- to 60-min observation periods under red light. Weevil behaviours measured were the following:

Presence on plant: This comprised the number of weevils on the plant. These were taken to be at risk of parasitism, whereas individuals absent from the plant were presumed less susceptible because they were often stationary or semi-buried in the soil.
Feeding: These were weevils on above-ground portions of plants with their heads down and rostrums penetrating the grass epidermis. When a weevil is feeding, the abdomen is slanted upwards, thereby exposing some inter-sternite membranes and anus, making them vulnerable to parasitoid oviposition (Phillips, 2002). Feeding coincided with presence on plant.

After the behavioural experiments were completed, all the leaves were removed from all the plants in all treatments and stored between microscope slides at −20°C for further feeding-damage analysis. The area (mm^2^) of the weevil feeding scars on each leaf segment was measured using the Olympus CellSens imaging software in conjunction with an Olympus SZX12 stereo microscope at 7.0-12.5 X magnification with an Olympus SC180 camera attached.

### Statistical Analysis

The behavioural variables analysed were the percentages of the 10 weevils in each replicate that were ‘present on plant’ and ‘feeding’. R version 3.6.3 (R Core Team, 2018) was used to analyse these data using a generalised linear mixed effects model with penalised quasi-likelihood estimation (GLMMPQL) to indicate the overall behavioural trends. The GLMMPQL was applied to all data collected in all observation periods and modelled correlation among repeated observations as first-order autoregressive random effects. The fixed effects of the GLMMPQL were grass cultivar (Manawa, Tama, and Samson), parasitoid presence/absence, and their interaction using binomial distributions through a logit link function. The GLMMPQL analysis was conducted using the glmmPQL function in the R package MASS (Venables and Ripley, 2002).

The behavioural responses within each of the four observation periods were investigated by fitting a generalised linear model (GLM) with a binomial error term using GenStat Release 20.1 (VSN International, 2019). The model terms for these analyses were replicate (a blocking factor), grass cultivar, parasitoid presence/absence trait responses, and their interaction. A logit (log-odds) link function was specified, and the dispersion parameter was estimated rather than fixed. A GLM was independently fitted for each behaviour in each of the four observation periods. Each GLM analysis estimated the means and least significant difference (LSD) at *P* < 0.05 for comparing each of the _6_C_2_ = 15 pairs of means. These LSDs were relatively large for comparing two large means, small for comparing two small means, and intermediate for comparing small and large means.

Weevil feeding-scar analyses were conducted using GenStat Release 20.1 (VSN International, 2019) and assumed that 10 weevils were unable to consume all the plant material available to them during a 6-h observation. Untransformed feeding-scar areas were analysed by the ANOVA with all assumptions met.

## Results

With reference to the results, the GLMMPQL analyses provided overall patterns using all the data available irrespective of observation period, whereas GLM focused on behaviour within each of the four observational periods.

### On-Plant Presence of *Listronotus bonariensis* in Response to *Microctonus hyperodae*

#### Cultivar Effect on Weevil On-Plant Response in the Presence of the Parasitoid vs. the Paired Absence Controls

**FIGURE 1:**
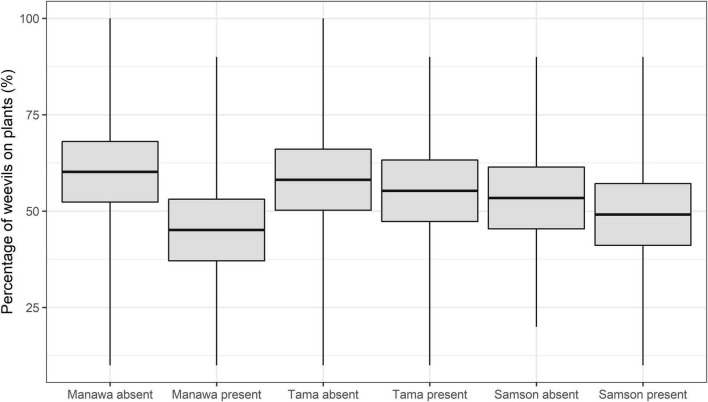
Mean percent of *Listronotus bonariensis* on *Lolium* grasses cv. Manawa, cv. Tama, and cv. Samson in the absence or presence of the parasitoid *Microctonus hyperodae*. The rectangles show the mean (central line) and 95% CL, and the tails show the range.

The GLMMPQL analysis showed that overall the observation intervals combined the percentage of weevils that were on the plants with *M. hyperodae* varied depending on the host grasses. Weevils on Manawa with the parasitoid had a significantly lower on-plant presence than those in the Manawa control (*t* = 2.4, df = 341, *P* < 0.02) (Figure 1). There were no significant differences in the on-plant presence of weevils on Tama (*t* = 0.5, df = 341, *P* > 0.05) or Samson (*t* = 0.7, df = 341, *P* > 0.05) irrespective of parasitoid presence or absence (Figure 1).

Similarly, the GLM analysis showed that on Manawa in the presence of the parasitoid, there were significantly lower percentages of weevils on plant in all four observation periods compared to the controls (df = 65 for observation periods 1-2, *P* < 0.01, *P* < 0.05, respectively, df = 66 for observation periods 3-4, *P* < 0.05, *P* < 0.01, respectively) (Table 1). Weevils on the Tama showed no significant on-plant response to the parasitoid. On Samson, there were significantly lower percentages of weevils on plant in the presence of the parasitoid (mean ± SEM: 42.2% ± 5.5%) compared to the control (58.7% ± 5.2%) only in observation period 4 (df = 66, *P* < 0.05) (Table 1).

**TABLE 1:** Behavioural experiment 1.

*Lolium* grass cultivar		Manawa (diploid hybrid)		Tama (tetraploid *L. multiflorum*)		Samson (diploid *L. perenne*)		LSD (5%)
				
*Microctonus hyperodae*		Absent	present	absent	present	absent	present	Range
Mean percent of *L. bonariensis* on-plant (± SEM)	Observation period 1	59.8 ± 4.9a	39.8 ± 4.8b	54.7 ± 4.5a	50.0 ± 4.6ab	50.0 ± 4.6ab	55.6 ± 4.7a	12.8 – 13.6
	Observation period 2	61.7 ± 4.7a	48.3 ± 4.7b	60.7 ± 4.4ab	59.7 ± 4.5ab	54.7 ± 4.5ab	49.6 ± 4.6ab	12.5 – 13.3
	Observation period 3	60.2 ± 4.5a	46.3 ± 4.5bc	58.7 ± 4.3ab	58.0 ± 4.3ab	54.2 ± 4.5abc	43.7 ± 4.4c	12.1 – 12.8
	Observation period 4	60.8 ± 5.4a	39.6 ± 5.3c	61.8 ± 5.3a	54.7 ± 5.2ab	58.7 ± 5.2a	42.2 ± 5.5bc	14.8 – 15.3

*Mean percentages (± SEM) of Listronotus bonariensis present on the three Lolium grass cultivars in the absence (control) or presence of Microctonus hyperodae.*

*Reading horizontally, values without letters in common differ significantly (P < 0.05). Letters are based on GLM least significant differences (LSD) at 5% (_6_C_2_ = 15 pairwise comparisons) with minimum and maximum LSDs given in the last column.*

#### Inter-Cultivar Comparisons of Weevil On-Plant Presence as Affected by the Parasitoid

The GLM analysis showed that in the presence of *M. hyperodae*, there were significantly fewer weevils on Manawa (39.6 ± 5.3%) than on Tama (54.7 ± 5.2%) in observation period 4 (df = 66, *P* < 0.05) (Table 1). Similarly, in the presence of the parasitoid, significantly more weevils were present on Tama (58.0 ± 4.3%) than on Samson (43.7 ± 4.4%) in observation period 3 (df = 66, *P* < 0.05) (Table 1).

### Feeding Responses of *Listronotus bonariensis* in the Presence of *Microctonus hyperodae* on the Three Grass Cultivars

The feeding responses by *L. bonariensis* to *M. hyperodae* on the three grass cultivars were found to be far more pronounced than their on-plant presence responses (Table 1).

#### Cultivar Effect on Weevil Feeding in the Presence of the Parasitoid vs. the Paired Absence Controls

**FIGURE 2:**
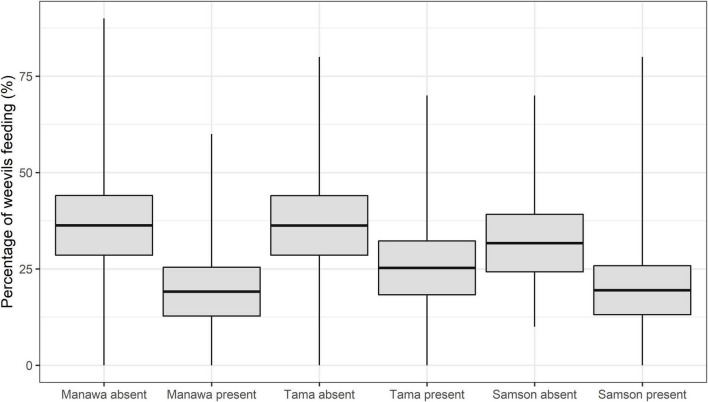
The mean percent feeding of the weevil *L. bonariensis* in the absence or presence of the parasitoid *M. hyperodae* on *Lolium* grasses cv. Manawa, cv. Tama, and cv. Samson. The rectangles show the mean (central line) and 95% CL, and the tails show the range.

The GLMMPQL analysis showed that, in the presence of *M. hyperodae*, weevil feeding was significantly reduced on all grass cultivars (*t* < 3.3, df = 341, *P* < 0.04) (Figure 2). However, this response was most apparent on Manawa (*t* = 3.2, df = 341, *P* < 0.002) (Figure 2).

Consistent with the GLMMPQL analysis, the GLM showed that weevils on Manawa fed less in the presence of the parasitoid compared to its control in all observation periods (df = 65 for observation periods 1-2, *P* < 0.05, *P* < 0.001, respectively (df = 66 for observation periods 3-4, *P* < 0.01, *P* < 0.01 respectively) (Table 2). Weevils on Tama fed significantly less in the presence of the parasitoid (mean ± SEM: 19.3% ± 4.4%) compared to the control (38.2% ± 5.7%) only in observation period 4 (df = 66, *P* < 0.05) (Table 2). Weevils on Samson fed less in the presence of the parasitoid compared to the control only in observation periods 3 and 4 (df = 66, *P* < 0.01) (Table 2).

**TABLE 2:** Mean percentages (± SEM) of *L. bonariensis* feeding on three *Lolium* grass cultivars in the absence (control) or presence of *M. hyperodae*.

*Lolium* grass cultivars		Manawa (Diploid hybrid)		Tama (Tetraploid *L. multiflorum*)		Samson (Diploid *L. perenne*)		LSD (5%)
				
*Microctonus hyperodae* treatment		Parasitoid absent	Parasitoid present	Parasitoid absent	Parasitoid present	Parasitoid absent	Parasitoid present	Range of values (min – max)
Mean percent of *L. bonariensis* feeding (± SEM)	Observation period 1	38.7 ± 5.0a	21.5 ± 4.1b	40.0 ± 4.6a	28.7 ± 4.3ab	34.7 ± 4.4a	30.3 ± 4.3ab	11.8 – 13.5
	Observation period 2	41.7 ± 4.9a	19.8 ± 3.8c	37.3 ± 4.4ab	30.9 ± 4.4abc	27.3 ± 4.1bc	20.5 ± 3.8c	10.7 – 13.2
	Observation period 3	34.9 ± 5.1a	17.4 ± 4.0bc	31.3 ± 4.7a	23.3 ± 4.3ab	29.0 ± 4.9ab	10.8 ± 3.3c	10.4 – 14.1
	Observation period 4	32.8 ± 5.4ab	14.5 ± 4.0c	38.2 ± 5.7a	19.3 ± 4.4bc	37.3 ± 5.4a	13.1 ± 4.0c	11.3 – 15.7

*Reading horizontally, means with no letters in common differ significantly (P < 0.05). Letters were assigned as in Table 1.*

#### Inter-Cultivar Comparisons of Weevil Feeding Responses as Affected by the Parasitoid

In the presence of the parasitoid, the GLM revealed only one significant difference between cultivars in the percentage of weevil feeding. In observation period 3, more weevils fed on Tama (23.3 ± 4.3%) than Samson (10.8 ± 3.3%) (df = 66, *P* < 0.05). However, fewer weevils also tended to feed on Manawa and Samson than Tama when the parasitoid was present (Table 2).

### Grass Consumption by *Listronotus bonariensis* in Response to *Microctonus hyperodae*

#### Cultivar Effect on Weevil Grass Consumption in the Presence of the Parasitoid vs. the Paired Absence Controls

**FIGURE 3:**
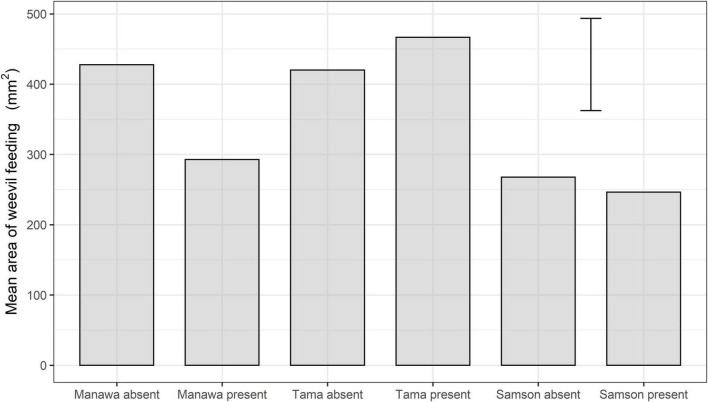
Mean feeding area (mm^2^) by the weevil *L. bonariensis* on Manawa, Tama, and Samson *Lolium* grasses in the absence or presence of the parasitoid *M. hyperodae*. The histogram shows the mean and an error term based on Fisher’s unprotected least significant differences (5%).

The ANOVA indicated that, on Manawa, the feeding area was significantly less in the presence of the parasitoid (mean ± SEM: 292.7 mm^2^ ± 46.5 mm^2^) than in its absence (424.6 mm^2^ ± 46.5 mm^2^) (df = 69, *P* < 0.05) (Figure 3). However, on Tama and Samson, there were no significant differences between parasitoid treatments (Figure 3).

#### Weevil Feeding Area and the Effects of Parasitoid Presence

In the presence of the parasitoid, the feeding scar area in Manawa was reduced significantly by 31% compared to the control. There we no significant differences in either Tama or Samson (Figure 3).

The feeding area was significantly higher on Tama than on Samson both in the absence (df = 69, *P* < 0.05) and the presence of the parasitoid (df = 69, *P* < 0.001) (Figure 3). In the absence of the parasitoid, there was significantly more weevil feeding on Manawa (424.6 mm^2^ ± 46.5 mm^2^) than on Samson (267.7 mm^2^ ± 46.5 mm^2^) (df = 69, *P* < 0.05).

## Discussion

### Host Plants Affecting Parasitoid-Avoidance Behaviours

This contribution has elucidated how host-plant species and/or their ploidy influences the extent of the selected-for *L. bonariensis*-avoidance behaviour in the presence of *M. hyperodae* (Shields, 2019). The diploid Manawa induced the greatest extent of avoidance behaviour by the weevils, which was manifested by lower on-plant presence and reduced feeding. Such behaviours were delayed on both the diploid Samson and the tetraploid Tama, with the latter being minor (Figures 1–3 and Tables 1, 2). These results therefore provide an explanation for the observed range of field and laboratory observations of weevil parasitism rates in pastures comprising different species and cultivars (Goldson et al., 2015; Goldson and Tomasetto, 2016; Tomasetto et al., 2017a,b).

#### Diploid Manawa

In the absence of the parasitoid, Manawa is a preferred host plant for the weevil, comparable with Tama (Goldson, 1982; Barker, 1989), thereby being characterised by similar weevil on-plant presence, feeding intensity (Figures 1–3 and Tables 1, 2), and ovipositional effort (Goldson, 1982; Barker, 1989). However, in the presence of the parasitoid, the weevil’s avoidance response to the parasitoid was significantly stronger on Manawa than on the other cultivars with greater reductions in on-plant presence, percentage of weevil feeding, and leaf area consumed (Figures 1–3 and Tables 1, 2). Significantly, in the 1990s, parasitism rates in Manawa were typically ca. 75%, in keeping with the other diploid *Lolium* grasses at that time (Goldson and Tomasetto, 2016). However, through parasitoid selection pressure causing enhanced weevil avoidance behaviour, parasitism levels have now declined to < ca. 46% in both the Manawa and several *L. perenne* cultivars (Goldson and Tomasetto, 2016; Tomasetto et al., 2017b).

These recently observed contrasting weevil behaviours on Manawa that now occur in the presence and absence of the parasitoid may well reflect the cultivar’s genetics in that it has characteristics of both *L. perenne* and *L. multiflorum.* In the breeding of this hybrid cultivar for livestock production, Corkill (1945) sought a medium tillered ryegrass that maximised the desirable features of palatability (e.g., less lignin and cellulose (Barker, 1989)) and the rapid establishment of *L. multiflorum*, while at the same time maintaining some degree of the density and persistency of *L. perenne*. Generally, it has been found that characteristics of high-performance pasture grasses for livestock production similarly support high weevil populations (e.g., Goldson, 1982; Barker, 1989; Ferguson et al., 2019).

#### Tetraploid Tama

Tama has maintained the same high parasitism levels that were originally found in the other grasses in the 1990s (Goldson and Tomasetto, 2016; Tomasetto et al., 2017c). This is reflected by the absence of any decline in weevil on-plant presence and limited feeding reduction when the parasitoid was present (Figure 1 and Table 2). Such a result would indicate that the weevils continue to be vulnerable to attack.

Unlike the other grasses discussed in this contribution, short-lived Tama plantings (and similar tetraploid *L. multiflorum* varieties) are not widespread (B.R. Belgrave, Grasslanz Technology Ltd., pers. comm.). This therefore may limit the potential for *L. bonariensis* to respond to selection pressure exerted by the *M. hyperodae* on Tama due to the cultivar’s relative scarcity. Further, the preference of weevils for Tama and other short-lived tetraploid *L. multiflorum* cultivars compared to diploid and other tetraploid cultivars (Goldson, 1982; Barker, 1989) could also work against any parasitoid-avoidance behaviour due to Tama’s superior nutritional quality (Charlton and Stewart, 1999; Sattler et al., 2016). These attractive short-lived tetraploid qualities include higher cell content of water (Sugiyama, 2006), nitrogen (Gaynor and Hunt, 1982), and water-soluble carbohydrates (Vartha and Bailey, 1974; Aderibigbe et al., 1982).

#### Diploid Samson

*Lolium perenne* pastures such as Samson are distributed across New Zealand and are favoured by farmers due to their persistence arising from their continuous production of secondary tillers (O’Connor et al., 2020). As mentioned above, in the 1990s, parasitism levels were often around 75% (Barker, 2013; Goldson and Tomasetto, 2016); however, these have now reduced to as little as ca. 14% in the central North Island and ca. 32% in central South Island regions of New Zealand (Shields et al., 2022). In keeping with these results, it was found that when exposed to the parasitoid, weevils on Samson showed similar responses to weevils on those on Manawa, though to a lesser extent (Figure 2 and Tables 1, 2). Possibly, the behavioural responses on Samson were less obvious than on Manawa because, in general, less feeding was observed (Figure 3), probably due to *L. perenne* having higher cellulose and sclerenchyma cell content (Evans, 1964, 1967).

### Varying Effects of Ploidy on Parasitism Rates

This study has highlighted an effect on *L. bonariensis* parasitoid-avoidance behaviour and parasitism rates based on the influence of tetraploidy in *L. multiflorum* such as Tama. Unlike the other grasses, there was minimal weevil parasitoid-avoidance behaviour and no parasitism decline (e.g., Goldson et al., 2015; Goldson and Tomasetto, 2016; Tomasetto et al., 2017a). Conversely, reduced parasitism rates have indeed been found in diploid *L. multiflorum* to a similar extent to that found in both diploid and tetraploid *L. perenne* (Goldson and Tomasetto, 2016; Tomasetto et al., 2017b). As well as lack of evolved behaviour on Tama (see above), a possible explanation for the observed weevil’s ongoing presence and feeding is that tetraploidy enhances characteristics (Charlton and Stewart, 1999; Sattler et al., 2016) favourable to the weevil (Barker et al., 1989), as such higher nitrogen content (Gaynor and Hunt, 1982). There may also be additional plant characteristics that contribute to the weevil’s behaviour on tetraploid *L. multiflorum* such as plant volatiles, but this was beyond the scope of this contribution.

The weevil’s parasitoid-avoidance pattern was found to be very different in the case of the hybrid Manawa grass. Here, in the parasitoid’s absence, the weevil exhibits behaviour similar to that of Tama. Conversely, in the parasitoid’s presence, the weevil shows all of the characteristics of those found on diploid *Lolium* plants. It is interesting to surmise that such departure from the pattern above could be the result of the hybrid having genetic components of both *L. perenne* and *L. multiflorum*. Notably though, it is unlikely that this ‘perennial and Italian ryegrass’ cross would have comprised any polyploidy as these typically have low seed germination rates (Griffiths et al., 1971).

## Conclusion

This study is one of the few that has demonstrated how evolved parasitoid-avoidance behaviours of a weevil pest species have varied according to the effect of plant species and cultivars. These findings also accommodate the original observations in 1990s that all pasture grasses had similar levels of parasitism and how varying selection pressures according to the grass type has now led to uneven levels of parasitism (Goldson and Tomasetto, 2016). Such information will provide insight into the management of successful importation biological control agents, particulary in broad-acre applications including pastures.

## Data Availability Statement

The raw data supporting the conclusions of this article will be made available by the authors, without undue reservation upon request.

## Author Contributions

MS, SG, SW, and CP conceived the ideas and designed the study. MS conducted the experiments. MS, SW, and SG collected the data. MS, CV, and CP analysed the data. MS and SG led the manuscript writing. All authors contributed to the drafts and gave final approval for publication.

## Conflict of Interest

SG, CP, and CV were employed by AgResearch Ltd. The remaining authors declare that the research was conducted in the absence of any commercial or financial relationships that could be construed as a potential conflict of interest.

## Publisher’s Note

All claims expressed in this article are solely those of the authors and do not necessarily represent those of their affiliated organizations, or those of the publisher, the editors and the reviewers. Any product that may be evaluated in this article, or claim that may be made by its manufacturer, is not guaranteed or endorsed by the publisher.
